# Reconciling Variability in Multiple Stressor Effects Using Environmental Performance Curves

**DOI:** 10.1111/ele.70065

**Published:** 2025-01-17

**Authors:** Hebe Carmichael, Ruth Warfield, Gabriel Yvon‐Durocher

**Affiliations:** ^1^ Environment and Sustainability Institute University of Exeter Penryn UK; ^2^ Department of Geography, Faculty of Science, Environment and Economy University of Exeter Exeter UK

**Keywords:** environmental change, multiple stressors, performance curves

## Abstract

Understanding the effects of multiple stressors has become a major focus in ecology and evolution. While many studies have investigated the combined effects of stressors, revealing massive variability, a mechanistic understanding that reconciles the diversity of multiple stressor outcomes is lacking. Here, we show how performance curves can fill this gap by revealing mechanisms that shape multiple stressor outcomes. Our experiments with 12 bacterial taxa, demonstrate that additional stressors alter the shape of temperature, pH and salinity performance curves. This leads to changes in stressor interaction outcomes—for example, shifts between additive, antagonistic or synergistic interactions—along gradients, revealing that small changes in a stressor along nonlinear performance curves can dramatically impact the stressor interaction. These findings help to explain the lack of generality found across multiple stressor studies and highlight how a performance curve approach can provide a more holistic view of multiple stressor interactions.

## Graphical abstract

Our experiments with 12 bacterial taxa, demonstrate that additional stressors significantly alter the shape of temperature, pH and salinity performance curves. This in turn leads to changes in emergent stressor interaction outcomes—for example, shifts between additive, antagonistic or synergistic interactions—along gradients, revealing that small changes in the level of a stressor along nonlinear performance curves can dramatically impact the assigned stressor interaction.

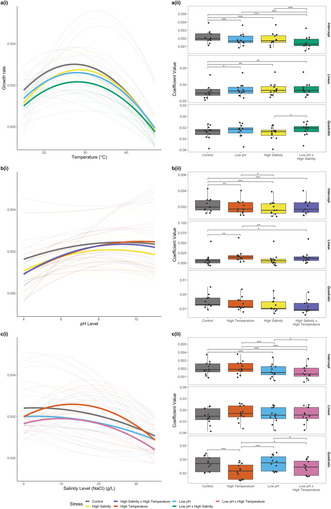

## 1 Introduction

Ecosystems are increasingly threatened by a multitude of environmental stressors (or ‘drivers’) from anthropogenic activities including climate change, chemical pollution and acidification (Pörtner et al. 2021). These stressors rarely act in isolation and their combined effects can be both complex and unexpected. In the last 20 years, multiple stressor research has grown rapidly, with studies aiming to understand and predict the way in which stressors interact to effect organism performance (Orr et al. 2020). Numerous experiments have yielded massive variability in the outcomes of multiple stressor interactions and have exposed significant gaps in our mechanistic understanding. Consequently, predicting the ecological outcomes of ecosystems exposed to multiple stressors is a major challenge that limits environmental management (Darling and Côté 2008; van Moorsel et al. 2023).

Typically, multiple stressor research focuses on pairs of stressors in a 2 × 2 factorial design. These designs treat continuous variables as discrete (e.g., ‘control’ vs. ‘stressful’ temperatures) and then commonly use additive (or multiplicative) null models to predict stressor interactions based on the sum (or product, if multiplicative) of their individual effects (Burgess et al. 2021; Collins, Whittaker, and Thomas 2022; Darling and Côté 2008; Jackson et al. 2016; Kreyling et al. 2018). Deviations from this predicted additive effect are broadly categorised as antagonistic (less than additive) or synergistic (greater than additive) or in some cases a dominance model may show that the effects of one stressor outweigh that of another (dominance effect) (Morris et al. 2022). While meta‐analyses have attempted to draw general patterns from multistressor studies, there is a striking lack of clear, observable trends in stressor interactions (Côté, Darling, and Brown 2016). Some have concluded that additive (Stockbridge, Jones, and Gillanders 2020) or synergistic (Burkepile and Hay 2006; Crain, Kroeker, and Halpern 2008; Harvey, Gwynn‐Jones, and Moore 2013; Przeslawski, Byrne, and Mellin 2015) interactions are most prevalent, while others suggest that antagonistic (Jackson et al. 2016; Lange et al. 2018; Tekin et al. 2020) or even dominance (Morris et al. 2022) effects are more common. This variability limits our ability to predict the effects of multiple stressors and suggests that a more in‐depth, mechanistic understanding of stressor interactions is required.

**FIGURE 1:**
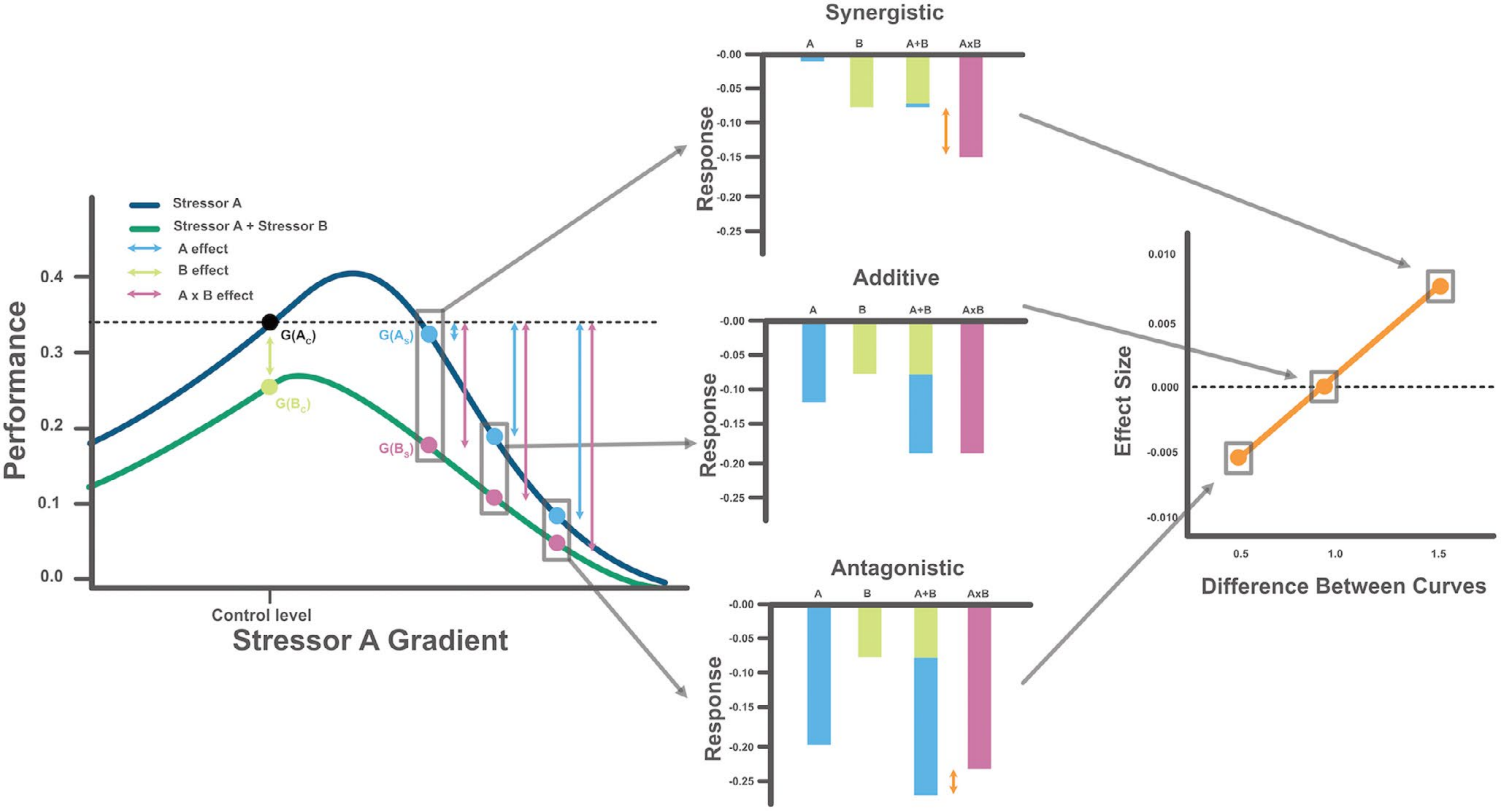
Stressor interactions change along gradients when performance curves are nonlinear. The left panel shows theoretical performance curves for stressor A in the presence and absence of stressor B. The black dot represents the performance of the organism in ‘control’ conditions, G(A_C_), and the light green dot is the performance in the presence of stressor B under control conditions of stressor A (G(B_C_)) (single stressor effect). The light blue dots, G(A_S_), and arrows represent the single stressor effect of stressor A along the gradient compared to the control, while the purple dots, G(B_S_), and arrows represent the combined effects of stressor A and B along the gradient compared with the control. The middle panels show the organism responses to stressors A and B relative to the control for different levels along the gradient. The bars labelled A + B represent the predicted additive response for A and B combined whereas the purple bars (A × B) show the observed response. The choice of stress levels along the gradient can alter the deduced interaction when stressors act in a nonlinear manner. Additive, synergistic and antagonistic interactions may all be observed between the same stressor combinations depending on which stressor levels are chosen. The right panel illustrates the relationship between the difference among the performance curves shown in the left panel (i.e., difference between the blue and purple points) and the effect size, defined as the difference between the observed and predicted additive response (indicated by the orange arrows in the middle panel). As the difference between curves increases, the effect size also increases. See Equation (2) and S1.5 for further description of the mathematical notation.

In recent years, there has been an increase in attention surrounding the use of performance curves (also referred to as response norms, reaction norms, dose–response curves, stressor–response functions) to understand multiple stressor interactions (Boyd et al. 2018; Collins, Whittaker, and Thomas 2022; Harley et al. 2017; Kreyling, Jentsch, and Beier 2014; Kreyling et al. 2018; van Moorsel et al. 2023; Orr et al. 2024; Pirotta et al. 2022; Rosenfeld et al. 2022; Schäfer et al. 2023; Segurado et al. 2022). This approach measures biological responses (e.g., population growth or mortality) to gradients of environmental parameters (Boyd et al. 2018). Many environmental parameters such as temperature (García et al. 2018), CO_2_ (Paul and Bach 2020), salinity (Rath et al. 2019) and pH (Bååth and Kritzberg 2015) tend to influence biological performance in a nonlinear, unimodal fashion, and only act as ‘stressors’ in a limited portion of the performance curve, away from an optimum. This nonlinearity in stressor responses could help explain why we see so much variation among multiple stressor studies, even when comparisons are made between the same stressor combinations (Stockbridge, Jones, and Gillanders 2020). For example, studies investigating two levels of each stressor in a factorial design, typically assume that biological performance changes linearly with variation in a stressor (e.g., when using additive null models), and hence multistressor interactions are constant regardless of the stressor levels chosen for experimentation. However, if biological responses to variation in stressors are nonlinear, then even slight changes in the stress levels chosen for experimentation in 2 × 2 factorial designs can have a large impact on the conclusions drawn on the direction and magnitude of stressor interactions (Figure 1). In contrast, adopting a gradient approach to analyse multistressor effects, by quantifying the shape of biological performance curves in response to individual and multiple stressors, would provide a more comprehensive understanding of the range of interaction outcomes that emerge when organisms encounter multiple environmental stressors that have nonlinear effects on biological performance (Boyd et al. 2018; Collins, Whittaker, and Thomas 2022; Kreyling et al. 2018; Pirotta et al. 2022; Segurado et al. 2022). For example, Figure 1 presents a hypothetical case where the multistressor outcome changes fundamentally along a stressor gradient, with the outcome switching from synergistic to antagonistic as the divergence between the control and stressor performance curves converge.

Here, we address these knowledge gaps using a tractable experimental design that employs a gradient approach to explore how multiple environmental stressors interact. We use aerobic heterotrophic bacteria as a model system, enabling high‐throughput measurement of biological performance (population growth rate) across gradients of three environmental variables (temperature, pH and salinity) in 12 bacterial taxa. We then explore how additional stressors (both single and pairs of stressors) alter the shapes of these performance curves and subsequently, the outcomes of the multistressor interactions.

## 2 Methods

### 2.1 Bacterial Taxa

The experiments were conducted using 12 freshwater heterotrophic bacterial taxa originally isolated from Icelandic geothermal pools (García et al. 2018) and experimental mesocosms in Dorset (Schaum et al. 2017). Taxa were identified using 16S rRNA sequencing and were identified as: *Curtobacterium* spp., *Microbacterium* spp. and *Pedobacter* spp., for the Dorset samples and as: *Aeromonas* spp., *Chromobacterium* spp., *Chryseobacterium* spp., *Erwinia* spp., *Janthinobacterium* spp., *Pseudomonas anguilliseptica* , *Pseudomonas fluorescens* , *Serratia* spp. and *Yersinia* spp. for the Icelandic samples.

### 2.2 Stressor Performance Curves

All experiments were conducted in 96‐well plates containing three replicates per taxa for each environmental condition. In all cases, bacteria were first grown in 48‐well plates in LB medium at 20°C for approximately 72 h immediately after coming out of the −70°C freezer. From here, bacteria were then transferred into R2 media and acclimated to the various stressful conditions (see below) for 24 h in 48‐well plates and the optical density (OD) was then measured using a Thermo Scientific Varioskan Lux multimode microplate reader to obtain the total bacterial biomass in OD_600_. To begin the experiment, samples were diluted back to a common starting density in the corresponding media to ensure growth rates could be compared and were then placed into 96‐well plates in incubators set at the corresponding temperatures. Bacterial biomass was measured via optical density (OD_600_) every 2 h for 12 h and then approximately every 4 h until carrying capacity was reached, providing us with a biomass time series. OD_600_ of 96‐well plates was measured using a Thermo Scientific Multiskan Sky Microplate Spectrophotometer standard plate reader at 600 nm. The specific conditions for each stressor gradient are given in S1.1 of the Supporting Information.

### 2.3 Statistical Analysis

All statistical analysis was carried out in *R* version 4.3.1 (R Core Team 2023). Growth rates were derived by fitting the Gompertz model (Gompertz 1997) to measurements of *OD*_600_ through time (see S1.2 of the Supporting Information for details of this model).

We used a quadratic model to capture the nonlinear stressor performance curves and fitted this model to the empirical data using a linear mixed‐effects modelling framework. The models were fitted using the *lmer* function from the lme4 package in *R* (Bates et al. 2015) and the coefficients of the quadratic model were introduced using the *poly()* function, which evaluates orthogonal polynomials over the range of the independent variable, x, and facilitates assessing the proportion of the variance in the dependent variable explained by each of the coefficients in the quadratic model (Bates et al. 2015). This approach has several advantages. First, the quadratic model is a simple and general unimodal function. Our aim in this analysis was to capture the essence of the unimodal environmental performance curves for each of the three gradients (temperature, pH and salinity) using a single model which enabled objective comparisons of the multistressor effects on curve shape. The quadratic model allowed us to achieve this. Second, the quadratic model has the advantage that it can be fitted to the data via a more efficient and flexible *linear* mixed‐effect model approach because the model is linear in the coefficients (Bates et al. 2015). The models took the form:

(1) $$r{\left(x\right)}_{i}=\bar{\beta_{0}}+\varepsilon_{0i}+\left(\bar{\beta_{1}}+\varepsilon_{1i}\right)x+\left(\bar{\beta_{2}}+\varepsilon_{2i}\right)x^{2}$$

where $r{\left(x\right)}_{i}$ is the growth rate derived from fitting the Gompertz model at stressor value, x, within the *i*‐th taxon, $\bar{\beta_{0}}$ is an average across taxa for the intercept, $\bar{\beta_{1}}$ is an average across taxa for the linear coefficient, $\bar{\beta_{2}}$ is an average across taxa of the quadratic coefficient. We expect estimates of $\beta_{0}$, $\beta_{1}$ and $\beta_{2}$, to vary among taxa, which we account for by treating the coefficients as random variables with averages of $\bar{\beta_{0}}$, $\bar{\beta_{1}}$ and $\bar{\beta_{2}}$ and deviations among taxa from these averages as $\varepsilon_{0}$, $\varepsilon_{1}$ and $\varepsilon_{2}$, respectively. The parameters of the quadratic model have no *a priori* mechanistic biological basis, instead the quadratic model captures the phenomenological unimodal shape of the environmental performance curve. The coefficients of the quadratic model can be interpreted as follows; intercept term, $\beta_{0}$, captures the elevation of the curve on the *y*‐axis, the linear term, $\beta_{1}$, determines the position of the curve along the stressor gradient, x, and the quadratic coefficient, $\beta_{2}$, determines the width of the curve (see Figure S1). Note, in our analytical approach, uncertainty in the estimation of $r{\left(x\right)}_{i}$ is not propagated into this analysis of the performance curve, as is common across the vast majority of studies that quantify performance curves based on biological rates (Bååth and Kritzberg 2015; García et al. 2018; Paul and Bach 2020; Rath et al. 2019).

We fitted Equation (1) to the performance curves for all three environmental variables in isolation as well as to performance curves quantified in the presence of each of the other variables under stressful conditions in all two‐ and three‐way combinations. We carried out three separate analyses, one for each type of performance curve—that is, temperature, pH and salinity. To compare the shapes of the performance curves under scenarios of multiple stressors, we included a fixed factor, ‘stress’ into Equation (1) which had levels of ‘control’ (i.e., with no additional stressor), ‘pH’, ‘salinity’ or ‘temperature’ (i.e., in presence of a single additional stressor) and ‘pH × salinity’, ‘pH × temperature’ or ‘salinity × temperature’ (i.e., in the presence of two additional stressors). To determine the best fitting model, we fitted all possible combinations of models using maximum likelihood, starting from the most complex model (i.e., the quadratic mixed‐effects model with interactions between the stress factors on all parameters with random effects) to the simplest model (i.e., linear model including stress factor as a main effect without interactions). To assess which model best fit the data, we ranked all the models using Akaike information criterion corrected for small sample size (AICc) and selected the model with the lowest AICc value (Table S1). To assess pairwise differences in the model coefficients between the various levels of the ‘stress’ factor, we conducted pairwise comparisons using the *emmeans* package in *R* (Lenth 2023). This analysis provided a comprehensive exploration of how the introduction of additional stressors influences the positions, widths and heights of the curves.

### 2.4 Stressor Interactions—Additive Null Model and Hedge's *d*

To assess how the outcome of multistressor interactions vary across gradients of individual stressors, we applied an additive null model to predict combined stressor effects at each level along the gradients (see Section S1.3 in Supporting Information for details on the multiplicative approach). This was carried out for each taxon. The additive null model predicts the growth rate under the combined effects of multiple stressors (here two stressors, *A* and *B*) as

(2) $$G\left(A_{B}\right)=\left(G\left(A_{s}\right)–G\left(A_{c}\right)\right)+\left(G\left(B_{c}\right)–G\left(A_{c}\right)\right)+G\left(A_{c}\right)$$

where $G\left(A_{s}\right)$ is the growth in stressor *A* under the stress treatment, s, condition for stressor *A*, $G\left(A_{c}\right)$ is the growth in control conditions, c, and $G\left(B_{c}\right)$ is the growth in stressor *B* at the condition c of stressor *A* (see Figure 1). When a third stressor is added (three‐way interaction) then $G\left(C_{c}\right)–G\left(A_{c}\right)$ was added to the equation where $G\left(C_{c}\right)$ is the growth rate under stressor *C* at condition c, of stressor *A*. The growth in control conditions, $G\left(A_{c}\right)$, acts as a baseline state, enabling us to quantify the effect of additional stressors. This equation provides the predicted growth rate for the combined effects of multiple stressors. Since growth rate cannot be negative, any predictions below zero were set to zero to avoid a bias towards antagonism. This scenario is more likely at higher stressor intensities, where individual stressors may already have detrimental effects on growth. Introducing an additional stressor under these conditions, could therefore lead to a negative growth prediction.

We calculated additive predictions for every stressor level measured along the gradient. The control response and the effects of additional single stressors were extracted from the ‘control’ conditions (see Figure 1). For example, with the temperature gradients, control responses and the single stressor effects of low pH and high salinity were obtained at 20°C. The single stressor effects of temperature where then obtained at each temperature level measured, allowing additive predictions to be made at each level along the gradient. The same procedure was carried out with the pH and salinity gradients, where control and single stressor effects of additional stressors were taken at pH 7.2 and 0 g NaCl/L, respectively, and the single stressor effects for pH and salinity were taken at each level along the respective curves. Combined stressor effects were then extracted at each level along the gradients when additional stressors were present, for example for temperature gradients, the effects of salinity at each temperature along the gradient provided us with the combined effects of temperature and salinity, allowing us to assess how multi‐stressor effects change along the temperature gradient.

We used Hedge's *d* (Hedges 1981) as a standardised mean difference to calculate multistressor interaction effect sizes by comparing the additive null model prediction to the actual observed combined stressor effect for each level along the gradients following methods from Jackson et al. (2016) (Jackson et al. 2016)—see S1.4 in the Supporting Information for further details.

### 2.5 Changes in Interactions Along Gradients

Once interactions had been classified, we then calculated the frequency of each interaction among the 12 different taxa at each stress level. This allowed us to determine whether the way stressors interact varied by taxa and whether this then varied along stressor gradients.

We used generalised additive models (GAMs) to statistically determine whether the outcome of multistressor interactions (Hedge's *d*) changed along each of the stressor gradients for each of the stressor combinations and to assess whether relationships differed between taxa. In the most complex models, Hedge's *d* was modelled as a function of the stress gradient and a cubic regression spline was used to capture potential nonlinear relationships. To account for any potential variations among taxa, the smooth term was grouped by taxon identity, allowing slopes to vary by taxa. The addition of taxa identity as a model parameter meant that intercepts could also vary by taxa. A variable selection mechanism was implemented using the ‘select = TRUE’ parameter. This feature facilitated the preservation of influential main effects within the model while applying shrinkage to the smoothing function of the stress gradient towards zero in instances where a weak correlation with the outcome was detected (Marra and Wood 2011; Wood 2011). This selection process ensures the model places emphasis on variables demonstrating stronger associations. Model selection entailed fitting a range of models with intercepts and interaction terms removed to test hypotheses about the potential differences in stressor interactions among taxa and between stressor levels. Model selection was carried out by computing Akaike information criterion corrected for small sample size (AICc), where models with the lowest AICc values were then chosen (Table S5). GAMs were fitted using the *mgcv* package in *R* (Wood 2011).

## 3 Results

### 3.1 Nonlinearity in Stressor Responses

**FIGURE 2:**
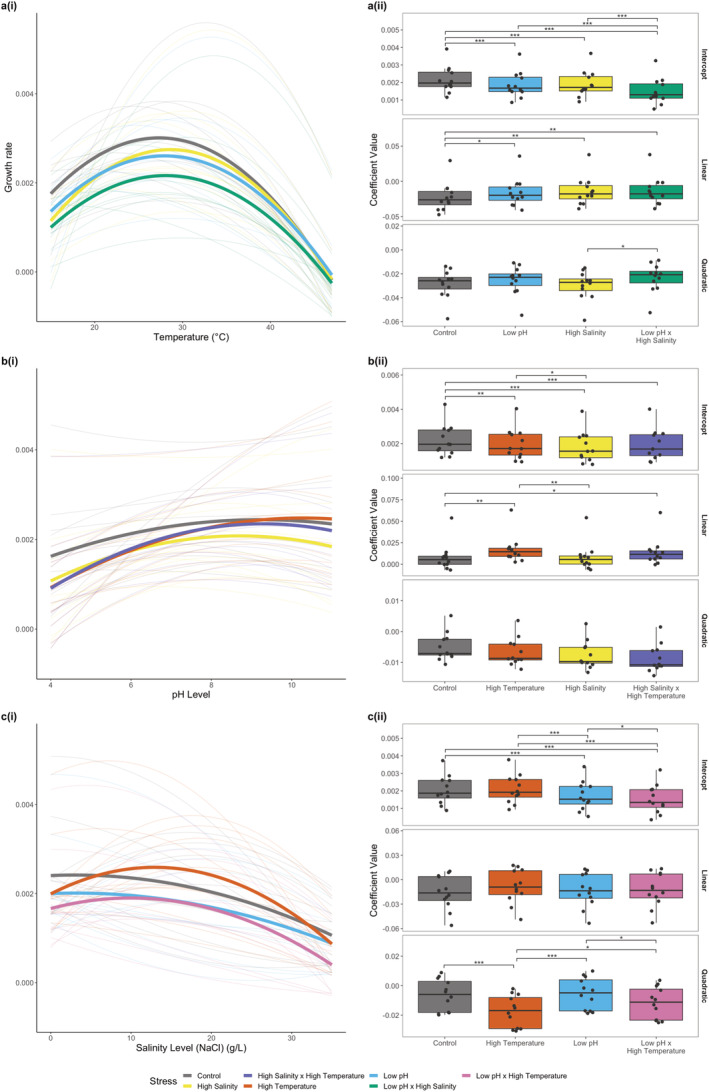
Effects of stressors on organism performance. (a(i), b(i), c(i)) Stressor performance curves for population growth rate across (a(i)) temperatures ranging from 15°C to 47°C, (b(i)) pH levels ranging from 4 to 11 and (c(i)) salinity levels ranging from 0 to 35 g NaCl/L. Thick, solid lines show the fixed effects of a quadratic model fitted via linear mixed effects, with taxa as a random effect for each stress gradient, using growth rate data from 12 bacterial taxa. Thin, semitransparent lines represent the performance curves for each of the individual taxa under the different stressor conditions derived from fixed and the random effects. Colours represent the addition of different stressor combinations where low pH = 5.5, high temperature = 38°C and high salinity = 20 g NaCl/L. Control conditions are 20°C, pH 7.2 and 0 g NaCl/L. Changes in curve height, shape and position with the addition of different stressors demonstrate how relationships between stressors vary along gradients. a(ii), b(ii), c(ii), Boxplots showing how additional stressors change the coefficients of the quadratic model fitted via linear mixed effects for (a(i)) temperature, (b(ii)) pH and (c(ii)) salinity performance curves. The intercept coefficient corresponds to curve height (e.g., the average growth rate across all levels of the stressor), the linear coefficient defines the curve position (left–right) and the quadratic coefficient quantifies the curve width. Black points highlight the variation in coefficients across the 12 taxa, derived from the fixed and random effects of the mixed effects model. Significance bars above the box plots represent pairwise comparisons, with the number of stars indicating the level of statistical significance (*p < 0.05, **p < 0.01, ***p < 0.001).

We measured changes in population growth rate across gradients of nine temperature levels (15°C–47°C), eight pH levels (pH 4–11) and eight salinity levels (0 g—35 g NaCl/L) separately to produce performance curves for each of these three environmental parameters across the 12 bacterial taxa. We also measured performance along these gradients in the presence of additional stressors in single and pairwise combinations to determine how multiple stressors affect species' performance (see Table S6 for stressor combinations). This enabled us to explore single, two‐way and three‐way stressor effects along each of the gradients. Levels of ‘stress’ were determined by quantifying regions of the performance curves where growth rates declined past the peak (see Section 2). To investigate general patterns across all 12 taxa, we fit a quadratic model to the data via linear mixed effects models, including taxon as a random effect for each stress gradient (Figure 2). We found that population growth rate varied with temperature, pH and salinity and did so in a nonlinear manner (Table S1). This was determined by comparing models including a quadratic term with reduced models that only had a linear term and finding that the inclusion of the quadratic term (i.e., nonlinearity) provided a better fit to the data for each for the three stressor performance curves (see Table S1, Figure 2).

### 3.2 Changes in Performance With Additional Stressors

Next, we investigated whether performance curves changed in the presence of additional stressors. We found significant changes in the shapes of the temperature, pH and salinity performance curves in all multistressor scenarios (Figure 2, Tables S2–S4). We compared the intercepts, linear terms and quadratic terms of the performance curves to determine how the height, position and width of curves changed in the presence of multiple stressors (see Figure S1, Figure 2). For the temperature performance curves, pairwise comparison tests of the intercepts revealed that low pH and high salinity significantly reduced taxa performance compared with control curves at 20°C (control temperature) (*t*(1801) = 4.33, *p* < 0.001, *t*(1801) = 3.75, *p* < 0.001 respectively), and the combination of these two stressors together further reduced performance to levels significantly lower than the single stressors alone (*t*(1801) = 5.70, *p* < 0.001, *t*(1801) = 6.26, *p* < 0.001, respectively) (Figure 2a, Table S2). We found similar patterns emerge in the intercepts of the pH and salinity performance curves, with additional stressors significantly reducing performance (Tables S3 and S4), and the combination of two stressors being significantly more detrimental than single stressors for salinity curves (Figure 2b,c, Table S4).

Pairwise comparisons of the linear terms and quadratic terms show that the shapes and positions of the curves varied significantly with the addition of stressors (Tables S2–S4). For example, in the pH performance curves (Figure 2b), the addition of temperature and temperature × salinity resulted in the position (linear term) of the curves shifting significantly to the right compared with the control (*t*(1642) = −2.97, *p* = 0.003, *t*(1642) = −2.01, *p* = 0.044 respectively), meaning that as pH levels decrease, the negative effects of these additional stressors became amplified. For the salinity curves (Figure 2c), we found that the addition of temperature stress significantly reduced curve width (quadratic term) compared with the control (*t*(1652) = 3.84, *p* < 0.001). This resulted in greater decreases in performance compared with the control in both low and high salinity conditions, yet for mid‐range salinity levels we found that the presence of high temperature enhanced organism performance.

In the temperature performance curves (Figure 2a), we not only saw the reduction in overall curve height, but we also observed a significant rightward shift in the position of the curve with the addition of all stressor combinations (pH: *t*(1801) = −2.38, *p* = 0.017, Salinity: *t*(1801) = −2.95, *p* = 0.003, pH × Salinity: *t*(1801) = −3.17, *p* = 0.002). The combination of these changes in the thermal performance curves resulted in performance levels converging across single and multistressor combinations at higher temperature levels.

These analyses reveal that general patterns emerge across diverse bacterial taxa in their responses to multiple stressors when looking across whole stressor gradients and taking a performance curve approach. Nevertheless, it is crucial to acknowledge the substantial variability among taxa in the shapes of their performance curves and multistressor responses (Figure S2). For example, Figure 2a(ii)–c(ii) illustrate the distribution of stressor effects on the parameters of the quadratic model and reveal that while there are significant effects of the single and multiple stressors on the parameters of the quadratic performance curves on average, substantial variability is present among the 12 bacterial taxa, highlighting the presence of both general patterns and taxon‐level idiosyncrasies, a common feature of ecological systems (Lawton 1999). To quantify the importance of accounting for taxon‐level variability in the shapes of the single and multistressor performance curves we fitted models to the dataset with and without the random effect terms that capture taxon‐level variability in the coefficients of the quadratic model. We found that accounting for taxon‐level variability in the coefficients of the quadratic model markedly enhanced the fit of the model to the data (Table S1, comparing models with and without random effects).

### 3.3 Stressor Interactions Along Gradients

To further investigate how nonlinear performance curves affect the outcome of interactions among multiple stressors, we determined multi‐stressor interaction types—for example, ‘additive’, ‘antagonistic’ or ‘synergistic’—for each taxon at every level of stress measured along the gradients (other than 20°C, pH 7.2 and 0 g NaCl/L which were used as control values) using the empirical growth rate data. Multistressor interactions were classified using Hedge's *d* to assess how the observed multistressor effect differed from the predicted additive null effect (Hedges 1981) (see Section 2). Negative Hedge's *d* values are associated with antagonistic interactions, positive values with synergistic interactions and any values where the confidence intervals cross zero are classed as additive (Figure S3).

**FIGURE 3:**
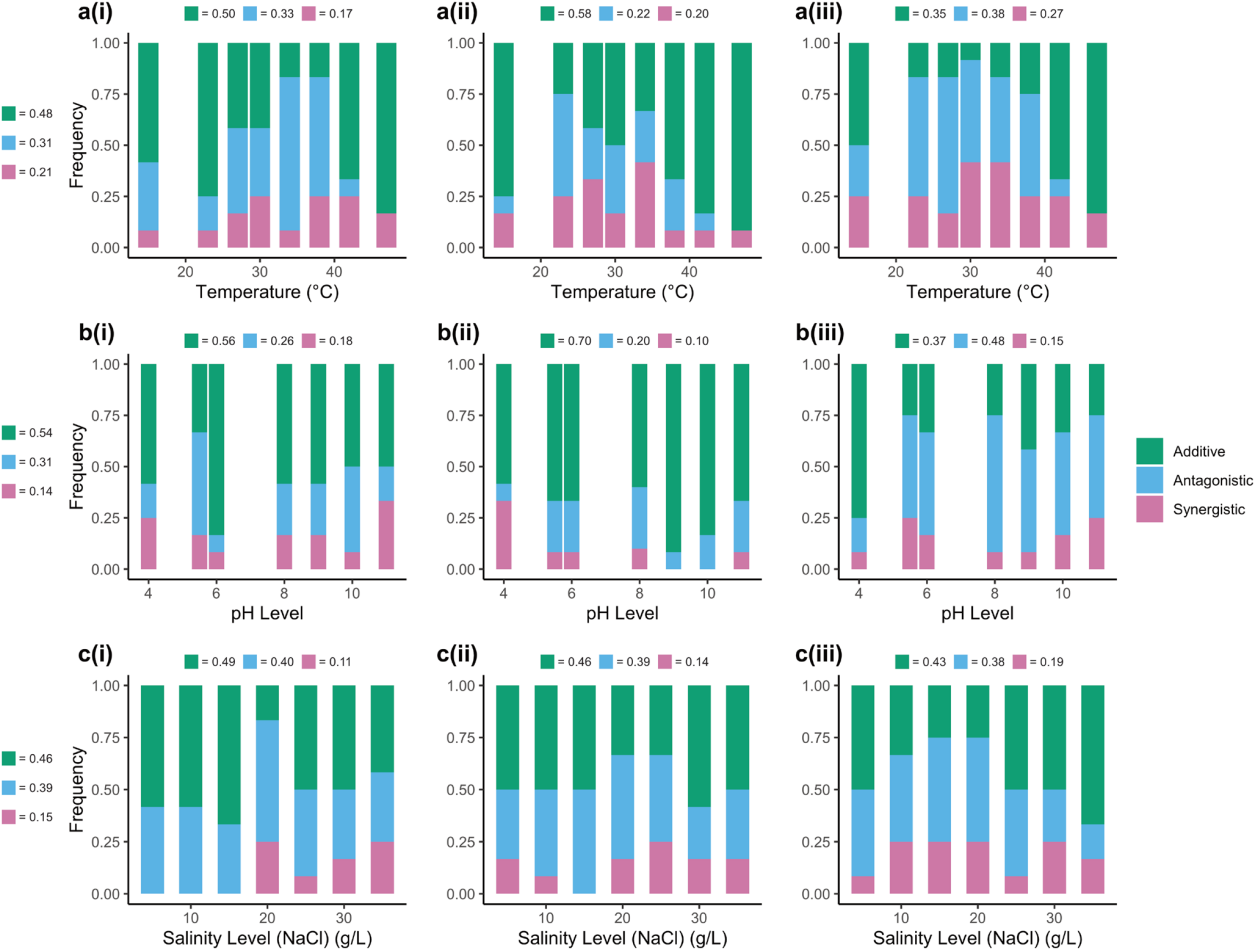
Frequency of stressor interactions across gradients. (a–c) The frequency of stressor interaction types (additive, antagonistic and synergistic) across (a) temperature, (b) pH and (c) salinity gradients for a total of 12 freshwater bacterial taxa. For temperature gradients, interaction type was determined for each taxon at every temperature level for (a(i)) temperature and high salinity (20 g NaCl), (a(ii)) temperature and low pH (pH 5.5) and (a(iii)) temperature, low pH and high salinity. For pH gradients, interaction type was determined for each taxon at every pH level for (b(i)) pH and high salinity, (b(ii)) pH and high temperature (38°C) and (b(iii)) pH, high temperature and high salinity. For salinity gradients, interaction type was determined for each taxon at every salinity level for (c(i)) salinity and high temperature, (c(ii)) salinity and low pH and (c(iii)) salinity, high temperature and low pH. Numbers above each panel show the total frequency of each interaction across all stressor levels measured. Numbers to the left‐hand side of each row show the overall frequency of interactions for each stressor gradient for all stressor combinations (i.e., total for each row).

We found substantial variation in multistressor outcomes among the 12 taxa, depending on where along the environmental gradient the assessment of multiple stressor interactions was conducted, with examples of antagonism, synergism and additivity for nearly all stress levels measured (Figure 3). The frequency at which these interactions occurred varied across the stressor gradients, implying that multistressor interactions vary depending on the stressor levels measured. For example, across temperature gradients (Figure 3a) synergism and antagonism tended to be most prevalent around the optimum temperature (~25°C–30°C), yet as temperatures became more stressful, additivity was more prevalent. Similar patterns were observed in the three‐way interactions for pH and salinity curves (Figure 3b(iii),c(iii)) where at low pH and high salinity levels, additivity increased, consistent with evidence from the performance curves, which reveal larger reductions in growth compared to the control under these levels (Figure 2, Figure S2). We also determined Hedge's *d* using the multiplicative null model (see Section 2) to ensure the results we observed were not dependent on the choice of null model. Consistent with the additive model, we found substantial variation in stressor interactions along the gradient, with multistressor outcomes being qualitatively similar across the two null models (Figure S4).

**FIGURE 4:**
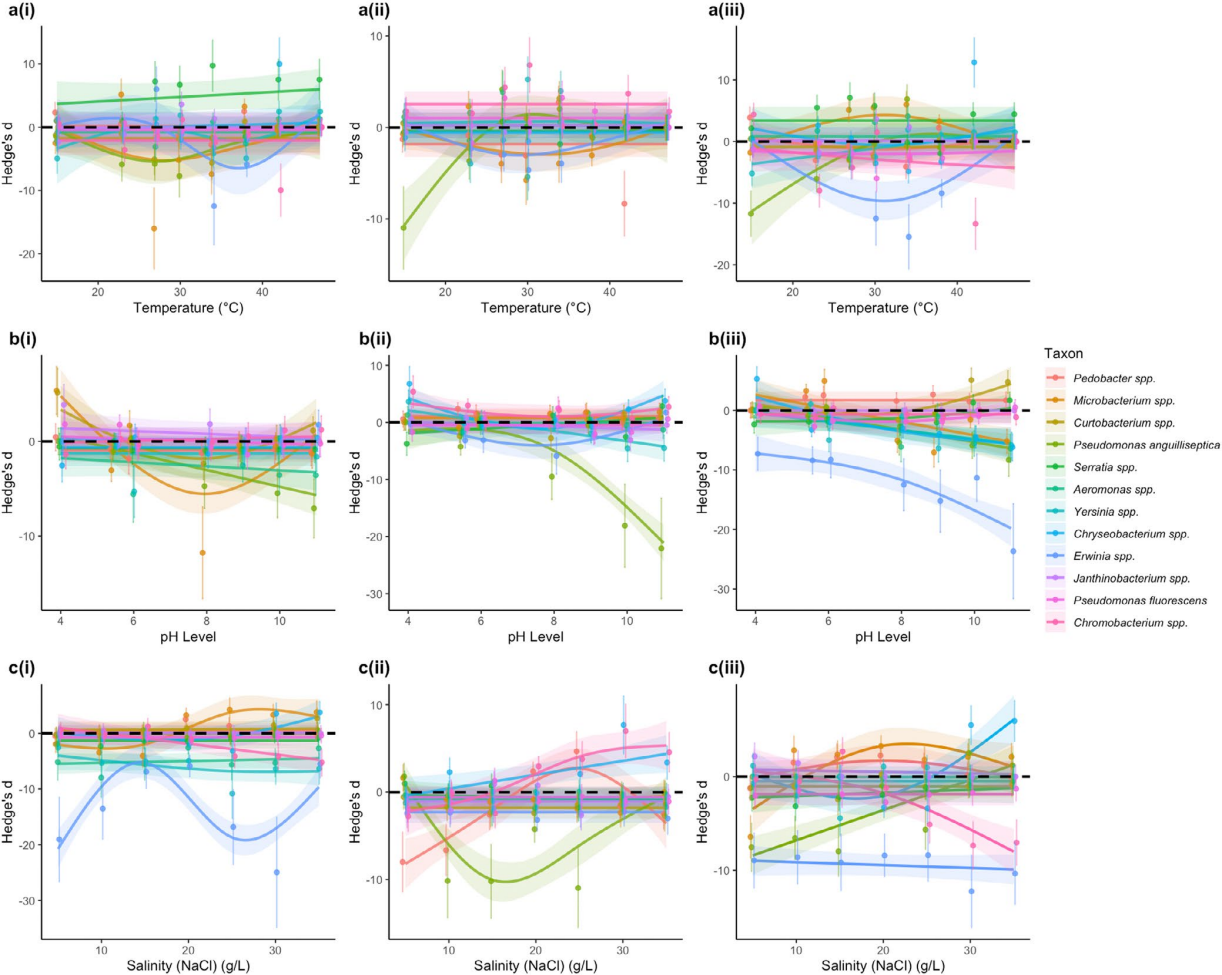
Taxonomic variation in stressor interactions along environmental gradients. (a–c) Changes in Hedge's *d* (interaction effect size for the additive null model) along (a) temperature, (b) pH and (c) salinity gradients for 12 bacterial taxa for different stressor combinations. Stressor combinations along temperature gradients were (a(i)) temperature and high salinity (20 g NaCl/L), (a(ii)) temperature and low pH (pH 5.5) and (a(iii)) temperature, high salinity and low pH. For pH, gradients were (b(i)) pH and high salinity, (b(ii)) pH and high temperature (38°C) and (b(iii)) pH, high salinity and high temperature. Combinations for salinity gradients were (c(i)) salinity and high temperature, (c(ii)) salinity and low pH and (c(iii)) salinity, high temperature and low pH. Coloured lines show the generalised additive model (GAM) fits for each stressor combination along the gradients, where slopes and intercepts are allowed to vary by taxon. Each colour represents a different taxon. Shaded areas represent the confidence interval around each of the GAM fits. Negative Hedge's *d* values are associated with antagonistic interactions, positive values with synergistic interactions and any values where the confidence intervals cross zero are classed as additive.

To explore how the outcome of the multistressor interactions changed along gradients of the stressors, we used generalised additive models (GAMs) to quantify the nature of the relationships between Hedge's *d* (i.e., the multistressor effect) and each stressor gradient for each taxon (Figure 4). We found that Hedge's *d* varied significantly across all stressor gradients and multistressor combinations and that the shapes of these relationships exhibited a wide variety of forms (Figure 4, Table S5). We also found that the way in which Hedge's *d* changed across stressor gradients varied significantly among taxa (Figure 4, Table S5). Across the different stressors, multistressor combinations and taxa, we observed both positive and negative linear trends, implying shifts from antagonism to synergism (and vice versa) along stressor gradients. We also found evidence for convex and concave relationships, implying that antagonism (concave) or synergism (convex) peaked at intermediate values along the stress gradient. Furthermore, we observed examples of fluctuations between all interaction types along the gradients, as well some examples where there was no relationship at all between the multistressor effect and the stressor gradient (Figure 4). These results serve to highlight the high variability and complexity in outcomes of the multistressor interactions that emerge from the nonlinear nature of environmental performance curves. They also reveal marked variation in multistressor outcomes between taxa and demonstrate substantial diversity in how these multistressor outcomes change along environmental gradients.

## 4 Discussion

Our results show that performance curves for 12 bacteria, over broad gradients in temperature, pH and salinity, were nonlinear and unimodal (Figure 2). While it is well known that single stressors often have nonlinear effects (Bååth and Kritzberg 2015; García et al. 2018; Rath et al. 2019), this is notable in the context of multistressor research which tends to assume linearity in responses (Collins, Whittaker, and Thomas 2022; Mack et al. 2022). If stress responses are nonlinear, as shown here, then relationships among multiple stressors and their interactions will depend on the relative shapes of performance curves along stressor gradients. The inclusion of additional stressor combinations also fundamentally altered the shapes of these environmental performance curves. Multiple stressors reduced the height of the performance curves resulting in lower average levels of performance as the number of stressors encountered increased. This general effect of reduced performance in a multistressor environment is consistent with a large body of prior experimental work (Barbosa et al. 2017; Christensen et al. 2006; Dinh, Janssens, and Stoks 2016; Jackson et al. 2016; Kholssi, Lougraimzi, and Moreno‐Garrido 2023; Morris et al. 2022). The inclusion of multiple stressors also however tended to make environmental tolerance curves narrower and shifted the location of the peak, resulting in lower performance at the extremes of the environmental gradients. These results highlight the generalities in inference that can be gained from taking a performance–curve approach across diverse taxa to the analysis of multistressor effects that would not be possible with a typical factorial ‘control’ versus ‘stress’ design.

We also found that the outcome of multistressor interactions—for example, ‘additive’, ‘antagonistic’ or ‘synergistic’—fundamentally changed along stressor gradients and that the nature of these relationships exhibited marked variability among the 12 taxa. These findings provide an explanatory basis for the massive range in multistressor outcomes observed in previous studies that adopt the typical ‘control’ versus ‘stressor’ factorial design (Côté, Darling, and Brown 2016). This is because small differences in the way experimental designs sample stressor levels along nonlinear environmental performance curves can result in massive differences in the outcome of multistressor interactions. Our findings support previous theoretical and simulation‐based studies suggesting that multistressor interaction outcomes vary depending on where on the environmental performance curves the sampling of ‘control’ and ‘stressful’ conditions occur (Collins, Whittaker, and Thomas 2022; Mack et al. 2022; van Moorsel et al. 2023; Segurado et al. 2022). This highlights the importance of choosing meaningful stressor levels in traditional factorial designs, such as known tipping points or accurate forecasts of future environmental conditions, which in most cases are unavailable (Collins, Whittaker, and Thomas 2022; Masson‐Delmotte et al. 2021).

Our results reveal that divergence in the shape of environmental performance curves when faced with additional stressors is directly linked to the outcome of multistressor interactions. For example, we found that the multistressor effect size is proportional to the difference between the performance curve measured under benign conditions (i.e., control) and the curve quantified under one or more additional stressors (see S1.5 and Figure S5 in the Supporting Information). Whether the divergence between the curves resulted in antagonism (negative), synergism (positive) or additivity (close to zero) depends on where the control condition was located on the performance curve (see S1.5). When the difference between curves was smallest at the control level then effect sizes were either zero or positive across other stressor levels along the gradient, resulting in the emergence of additive or synergistic interactions (see S1.5)—for example, the addition of high temperature along the pH gradient denoted by the triangles in Figure S5b (also see Figure 2b(i) to compare the control performance curve in grey with high temperature in orange). By contrast, when the largest difference between curves was observed at the control level, this led to negative effect sizes and hence additive or antagonistic interactions (see S1.5)—for example, the addition of low pH along the temperature gradient denoted by the triangles in Figure S5a (also see Figure 2a(i) to compare the control performance curve in grey with low pH in blue). These results reveal that when additional stressors alter the shape of environmental tolerance curves, nonadditive interactions emerge along the gradient and their direction and magnitude can be measured based on the difference between curves. This analysis shows that the multistressor effect size determined in any 2 × 2 factorial experiment represents a single point along a continuum of potential effect sizes that are linearly proportional to the magnitude of the difference between performance curves.

Variability in how different taxa respond to multistressor environments across stressor gradients adds another layer of complexity. Previous meta‐analyses have yielded mixed results regarding the most common type of multistressor interaction (Côté, Darling, and Brown 2016; Crain, Kroeker, and Halpern 2008; Jackson et al. 2016; Lange et al. 2018; Morris et al. 2022; Stockbridge, Jones, and Gillanders 2020). Our findings also reveal substantial variability in multistressor outcomes (Figures 3 and 4). We found that variability in multistressor outcomes emerged both *within* taxa along gradients in environmental stress and *between* taxa when assayed at the same environmental conditions (Figure 4). Such variability in multistressor outcomes is a direct result of nonlinear environmental performance curves (Figure 2), which mean that small changes in the level of an environmental variable along a gradient can manifest in large shifts in multistressor interaction outcomes when two nonlinear performance curves interact. We also found that whilst the frequency of multistressor interaction types varied between taxa along the gradients, additive interactions were generally the most prevalent when averaged across all stressor levels and combinations (Figure 3). This prevalence however was driven by a higher occurrence of additive interactions at extreme stressor levels, where taxa typically exhibit zero growth, resulting in additive predictions of no growth (see Section 2). Additionally, we observed a limited proportion of synergistic interactions across the gradients, which contrasts with findings from several previous meta‐analyses (Burkepile and Hay 2006; Crain, Kroeker, and Halpern 2008; Harvey, Gwynn‐Jones, and Moore 2013; Przeslawski, Byrne, and Mellin 2015). It is possible that the high occurrence of synergistic interactions reported in these studies could be influenced by publication bias, as has been suggested previously (Côté, Darling, and Brown 2016; Orr et al. 2020). However, it is important to acknowledge that our study used only one level of each additional stressor. In light of our findings on the impact of nonlinear performance curves on multistressor outcomes, it is highly likely that even slight adjustments to the ‘stress’ levels and the ‘control’ levels chosen in our experiment could produce qualitatively different outcomes. Furthermore, results may differ when exploring other organisms and environmental contexts (Kroeker, Kordas, and Harley 2017). This highlights the importance of studying a range of stressor levels and the need for future research to explore how these patterns manifest across different taxa and scenarios.

By shifting from typical two‐level factorial designs to a stressor gradient approach, our results show that a more mechanistic understanding of how stressors interact to produce emergent multistressor outcomes can be gained. Furthermore, the performance curve approach opens opportunities for the development of models, enabling predictions of responses to various environmental change scenarios. The use of a diverse range of taxa also provides the potential to scale up from population to community level predictions by using taxon‐level response traits to predict how communities composed of these taxa may respond to environmental changes (Collins, Whittaker, and Thomas 2022; De Laender 2018; Enquist et al. 2015; van Moorsel et al. 2023; Suding and Goldstein 2008; Thompson, MacLennan, and Vinebrooke 2018). Such models could provide crucial insights that are currently lacking. However, while our work establishes a foundation for understanding and predicting the impacts of multiple environmental stressors, significant challenges remain before these insights can be translated into actionable ecosystem management strategies. This includes conducting further performance curve studies across diverse trophic levels and ecological contexts, a very challenging task due to the scale and complexity that such experiments would require, especially with larger, slower‐growing organisms (Boyd et al. 2018). Furthermore, our results highlight the inescapable reality that multistressor outcomes exhibit massive variability both among taxa and within taxa along environmental gradients, meaning that developing general, mechanistic models to predict the emergent outcomes of multiple stressors is still a major challenge.

## Author Contributions

H.C. and G.Y.‐D. wrote the manuscript. H.C. and R.W. performed the experiments. G.Y.‐D. and H.C. designed the experiments. G.Y.‐D. conceived the study.

## Conflicts of Interest

The authors declare no conflicts of interest.

### Peer Review

The peer review history for this article is available at https://www.webofscience.com/api/gateway/wos/peer‐review/10.1111/ele.70065.

## Supporting information


Data S1.

## Data Availability

All data and code required to reproduce the analyses in this paper can be found at https://doi.org/10.5281/zenodo.14285702.
