# Supplementary material for: Reconciling Variability in Multiple Stressor Effects Using Environmental Performance Curves
Source: Ecol Lett. 2025 Jan 17;28(1):e70065. doi: 10.1111/ele.70065 (PMC11741915; doi:10.1111/ele.70065)
Supplement: Supplementary file 1 — Data S1. [file ELE-28-0-s001.pdf]

Supplementary Information for:

Reconciling variability in multiple stressor effects using environmental performance curves.

Hebe Carmichael[1], Ruth Warfield[1], Gabriel Yvon-Durocher[1, *]

[1] Environment and Sustainability Institute, University of Exeter, Penryn, United Kingdom, TR10 9FE.

[*] Corresponding author. E-mail: g.yvon-durocher@exeter.ac.uk

The file includes:
Section S1
Figs. S1 to S5
Tables S1 to S6

Section S1 – Supplementary Methods

S1.1 Stressor performance curves

Temperature gradient:

To understand how taxa respond to different stressful conditions across temperature gradients, they were placed into Percival incubators set to nine temperature levels (15°C, 20°C, 23°C, 27°C, 30°C, 34°C, 38°C, 42°C, 47°C) in control, low pH, high salinity or low pH x high salinity R2 medias (see Table S6 for levels).

pH gradient:

For pH gradients, taxa were placed in R2 medias of eight pH levels (4, 5.5, 6, 7.2, 8, 9, 10, 11) with either no additional stressors (control), high salinity, high temperature or a combination of high temperature and high salinity (see Table S6 for levels). pH levels were manipulated by adding either hydrochloric acid (HCl) for low pH levels or sodium hydroxide (NaOH) for high pH levels.

Salinity gradient:

Taxa were placed into R2 media of eight salinity levels (0g, 5g, 10g, 15g, 20g, 25g, 30g, 35g NaCl/L) with either no additional stressors (control), low pH, high temperature or low pH x high temperature (Table S6). Salinity levels were manipulated by adding sodium chloride (NaCl).

The levels of additional stressors were chosen using preliminary performance curve experiments to determine which levels were stressful to most taxa. For each stressor (temperature, pH and salinity), we determined the level of stress that resulted in a reduction in growth compared to the control laboratory conditions (20°C, pH7.2 and 0g NaCl – which reflect the conditions the taxa were isolated under) yet did not result in mortality for the majority of the taxa. For temperature and salinity, these were calculated from values above the optimum where growth declines rapidly and for pH were taken from values below the optimum (i.e. under more acidic conditions) where we observed the greatest decrease in performance across the levels measured.

S1.2 Deriving growth rate

Growth rates were derived by fitting the Gompertz model (Gompertz 1997) to measurements of $log_{10}OD_{600}$ through time, t (in minutes):

$$log_{10}OD_{600} = log_{10}n_0 + (log_{10}n_{max} - log_{10}n_0) \times e^{\left(-e^{1+r\times e^1 \times \left(\frac{lag-t}{(log_{10}n_{max} - log_{10}n_0)\times \ln(10)}\right)}\right)}$$

(Equation S1)

where $log_{10}n_0$ is the starting density, $log_{10}n_{max}$ is carrying capacity, r is the exponential growth rate (h^{-1}) and lag is the lag time in minutes. Model fitting was carried out using non-linear least squares regression using the *R* package *'nls.multstart'* (Padfield *et al.* 2020; Padfield & Matheson 2023). This involved running 500 iterations of the fitting process with

start parameters drawn from a uniform distribution and retaining the fit with the lowest Akaike Information Criterion (AIC) score.

S1.3 Stressor interactions – Multiplicative null model

In addition to the additive null model, we applied a multiplicative null model to predict the combined effects of multiple stressors at each level along the gradient. The multiplicative model assumes that the effect of combined stressors is the product of their individual effects relative to control conditions. Specifically, for two stressors, the predicted growth rate under the multiplicative model was calculated as:

$$\left(\frac{G(A_S)}{G(A_C)}\right) \times \left(\frac{G(B_C)}{G(A_C)}\right) \times G(A_C)$$

(Equation S2)

where $G(A_s)$ is the growth in stressor *A* under the stress treatment, s, condition for stressor *A*, $G(A_c)$ is the growth in control conditions, c, and $G(B_c)$ is the growth in stressor *B* at the condition c of stressor *A*. . For three stressors (i.e., a three-way interaction), the equation was extended to:

$$\left(\frac{G(A_S)}{G(A_C)}\right) \times \left(\frac{G(B_C)}{G(A_C)}\right) \times \left(\frac{G(C_C)}{G(A_C)}\right) \times G(A_C)$$

(Equation S3)

where $G(B_c)$ is the growth in stressor *C* at the condition c of stressor *A*. Hedge's *d* was then used to classify interaction types, applying the same method as for the additive null model but using predictions from the multiplicative model instead. This allowed us to assess whether patterns of interactions across gradients remained consistent when different null models were applied.

S1.4 Hedge's d

To calculate Hedge's *d*, the mean growth, standard deviation and sample size values were first extracted for each taxon at every level along the gradients and for every stressor combination for single stressors, two-way stressor combinations, three-way stressor combinations and the control. After calculating the additive prediction for all the single stressor combinations, Hedge's *d* was then calculated by comparing the predicted additive effect (X_p) with the actual observed effect (X_o) for stressors applied in combination:

$$Es_{Add} = \frac{X_o - X_p}{S} j$$

(Equation S4)

where *j* is a weighting factor based on the number of replicates (n), calculated as:

$$J = 1 - \frac{3}{4(n_o + n_p - 2) - 1}$$

(Equation S5)

S is the pooled standard deviation, calculated as:

$$S = \sqrt{\frac{(n_o - 1)(s_o)^2 + (n_p - 1)(s_p)^2}{n_o + n_p - 2}},$$

(Equation S6)

where the predicted standard deviation was calculated by pooling the standard deviation of the single stressor effects and the pooled sample size (n_p) was calculated by pooling the standard deviations of the single stressor treatments. The variance (V_{Add}) around each interaction effect was calculated as:

$$V_{Add} = \frac{n_o + n_p}{n_o n_p} + \frac{{Es_{Add}}^2}{2(n_o + n_p)}$$

(Equation S7)

The standard error was then calculated as:

$$SE_{Add} = \sqrt{V_{Add}}$$

(Equation S8)

and finally, confidence intervals were calculated as:

$$CI_{Add} = Z_{\alpha/2} \times SE_{Add}$$

(Equation S9)

where $Z_{\alpha/2}$ is the calculated value to get a significance level of 0.05 based on a t-distribution, therefore allowing us to obtain 95% confidence intervals.

We inverted the response direction ($\mp$) of the interaction effect sizes when the predicted effects were negative relative to the control. This allowed us to compare the strength of the effect size on an absolute scale irrespective of their directionality (Jackson *et al.* 2016; Piggott *et al.* 2015). As a result, an effect size of zero represents an exact additive effect, a negative effect size represents an antagonistic effect, and a positive effect size represents a synergistic effect. Multi-stressor interaction significance was assessed using 95% confidence intervals calculated around each effect size where interactions with confidence intervals crossing zero were classed as additive (Fig. S3).

S1.5 Linking performance curves to multi-stressor effects

The additive null model predicts the growth rate under the combined effects of multiple stressors (here two stressors, *A* and *B*) as

$$G(A_B) = (G(A_s) - G(A_c)) + (G(B_c) - G(A_c)) + G(A_c)$$

(Equation S10)

where $G(A_s)$ is the growth in stressor *A* under the stress treatment, s, for stressor *A*, $G(A_c)$ is the growth in control conditions, c, and $G(B_c)$ is the growth in stressor *B* at the condition

c for stressor A (see Fig. 1 for a diagrammatic representation). Using equation S10 we can derive a multi-stressor effect size as

$$ES = ((G(A_s) - G(A_c)) + (G(B_c) - G(A_c)) + G(A_c)) - G(B_s) \quad \text{(Equation S11)}$$

Where $G(B_s)$ is growth in stressor *B* at level s of stressor *A* – i.e. stressful conditions of both stressor *A* and stressor *B*. Equation S11 quantifies difference between the growth rate under the multi-stressor effect and the growth rate predicted if the effects of stressors *A* and *B* were additive. In this analysis the conditions, *c* and *s*, which determine the control and stress treatment levels for stressors *A* and *B*, are two arbitrary fixed points taken from the non-linear performance curves that characterise the effect of stressor *A* alone on growth rate, $G(A)$, and the effect of stressor *A* on growth rate under stressful conditions of stressor *B*, $G(A_{B_s})$. It can be shown that the multi-stressor effect size, ES, is proportional to the difference between the performance curves $G(A)$ and $G(A_{B_s})$, Δ_{AB}. The difference between curves *A* and *B* at stress treatment level, s, is given by

$$\Delta_{AB} = G(A_s) - G(B_s) \quad \text{(Equation S12)}$$

The expression for the multi-stressor effect size, Equation S11, can be simplified to

$$ES = G(A_s) - G(A_c) + G(B_c) - G(B_s) \quad \text{(Equation S13)}$$

Substituting Equation S12 into Equation S13 and simplifying terms gives

$$ES = \Delta_{AB} + (G(B_c) - G(A_c)) \quad \text{(Equation S14)}$$

Equation S14 shows that ES, is a linear function of Δ_{AB} with a slope of 1 and a constant $(G(B_c) - G(A_c))$. This analysis shows that the multi-stressor effect size determined in any 2x2 factorial experiment represents a single point along a continuum of potential effect sizes that is linearly proportional to the magnitude of the difference between performance curves, $G(A)$ and $G(A_{B_s})$ given by Δ_{AB}. Furthermore, equation S14 shows that the sign and magnitude of the multi-stressor effect size depends on where the control is located on the curves $G(A)$ and $G(A_{B_s})$. When the difference between curves is smallest at the control level, $\Delta_{AB} > (G(B_c) - G(A_c))$, then effect sizes are either zero or positive across other stressor levels along the gradient, resulting in the emergence of additive or synergistic interactions. By contrast, when the largest difference between curves was observed at the control level, $\Delta_{AB} < (G(B_c) - G(A_c))$, effect sizes are negative and hence additive or antagonistic interactions emerge.

To test this theory, we quantified the relationship between the multi-stressor effect size and the difference between the performance curves under a single versus multi-stressor environment we used the growth rates predicted from the aggregate performance curves derived from the fixed effects of the mixed-effects models (see Fig. 2). These data yield the average performance curve across the 12 taxa and are illustrative of the aggregate patterns across taxa. As predicted by Equation S14, we found that for all stressor gradients there was a significant positive, linear association between the additive stressor effect size and the

difference between the control performance curve and the performance curve under the stress condition (Fig. S5; Temperature: r^2 =1, p<0.0001, pH: r^2 =1, p<0.0001, Salinity: r^2 =1, p<0.001). Furthermore, the slopes of the relationships between ES and Δ_{AB} were all indistinguishable from unity, consistent with Equation S14.

Supplementary Figures:

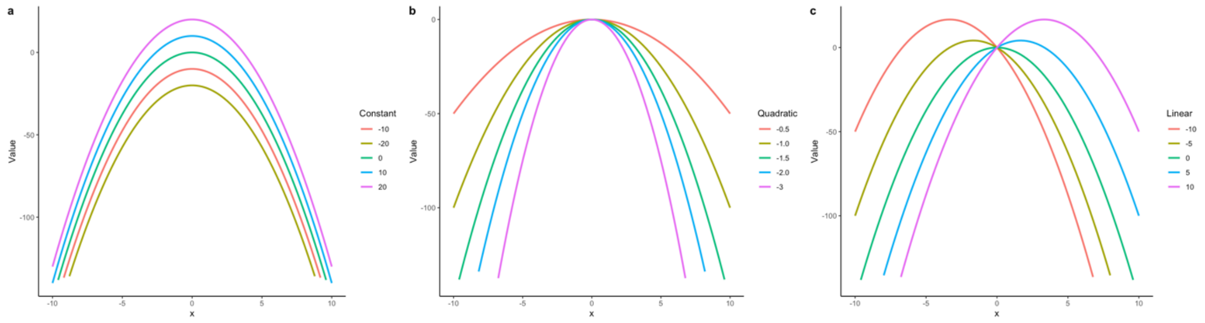

Figure S1. Changes in curve height, shape and position with different quadratic terms. The quadratic equation is made up of the constant (intercept) term (β_0), linear term ($\beta_1 x$) and the quadratic term ($\beta_2 x^2$), and has the form $r(x) = \beta_0 + \beta_1 x + \beta_2 x^2$. Panel **a** shows how changing the constant alters curve height as the y intercept is always equal to the constant and panel **b** shows how changing the quadratic term changes the width of the curve. Panel **c** shows how changing the linear term effects the curve position. When the constant is set to 0, decreases in the linear term away from 0 shift the curve to the left and up whilst increases from 0 shift it to the right and up. The upwards shift is due to the constant remaining the same here.

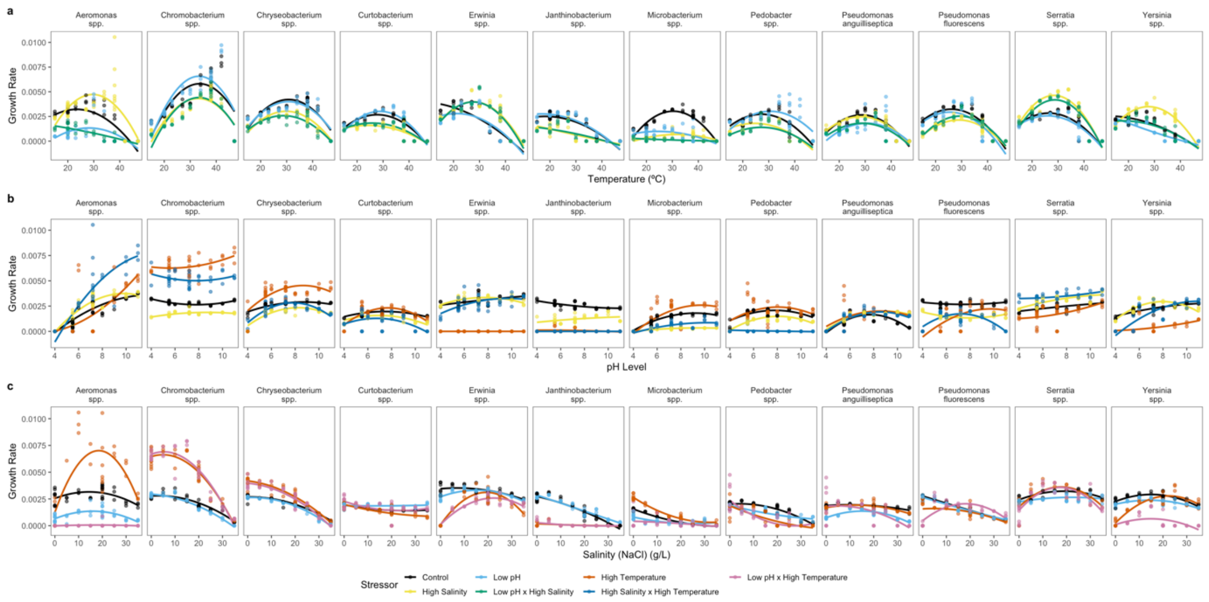

Figure S2. Variability in stress responses between 12 bacterial taxa. a-c, Stressor performance curves for population growth rate across (**a**) temperatures ranging from 15 to 47 °C, (**b**) pH levels ranging from 4 to 11 and (**c**) salinity levels ranging from 0g to 35g NaCl per litre. Quadratic curves were fit via a linear model for each of the 12 bacterial taxa with the stress gradient (e.g. temperature for (**a**)) and additional stressors as model parameters. Colours represent the addition of different stressor combinations where low pH = 5.5, high temperature = 38°C and high salinity = 20g NaCl per litre. Control conditions are 20°C, pH 7.2 and 0g NaCl per litre.

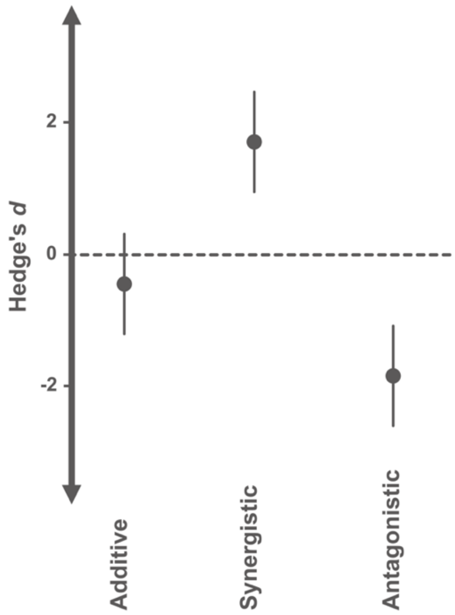

Figure S3. Hedge's *d* for determining stressor interactions. Theoretical interaction effects of 'observed' multiple stressors in combination relative to their predicted additive response (=0). Positive (greater than zero) Hedge's *d* values represent synergism, negative values (less than zero) represent antagonism and any values where the confidence intervals cross zero are classed as additive.

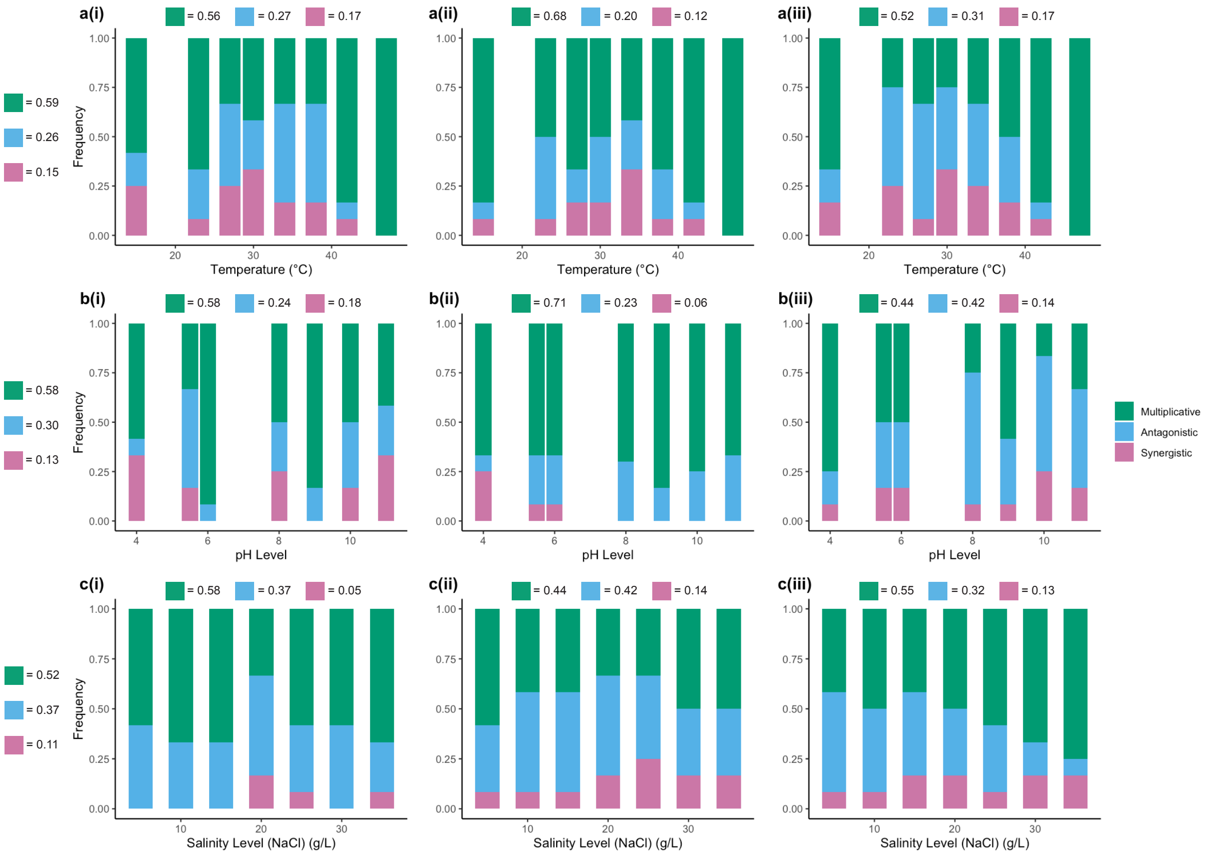

Figure S4. Frequency of stressor interactions across gradients (multiplicative). a-c, The frequency of stressor interaction types (multiplicative, antagonistic and synergistic) across (**a**) temperature, (**b**) pH and (**c**) salinity gradients for a total of 12 freshwater bacterial taxa. For temperature gradients, interaction type was determined for each taxon at every temperature level for (**a(i)**) temperature and high salinity (20g NaCl), (**a(ii)**) temperature and low pH (pH 5.5) and (**a(iii)**) temperature, low pH and high salinity. For pH gradients, interaction type was determined for each taxon at every pH level for (**b(i)**) pH and high salinity, (**b(ii)**) pH and high temperature (38°C) and (**b(iii)**) pH, high temperature and high salinity. For salinity gradients, interaction type was determined for each taxon at every salinity level for (**c(i)**) salinity and high temperature, (**c(ii)**) salinity and low pH and (**c(iii)**) salinity, high temperature and low pH. Numbers above each panel show the total frequency of each interaction across all stressor levels measured. Numbers to the left-hand side of each row show the overall frequency of interactions for each stressor gradient for all stressor combinations (i.e. total for each row).

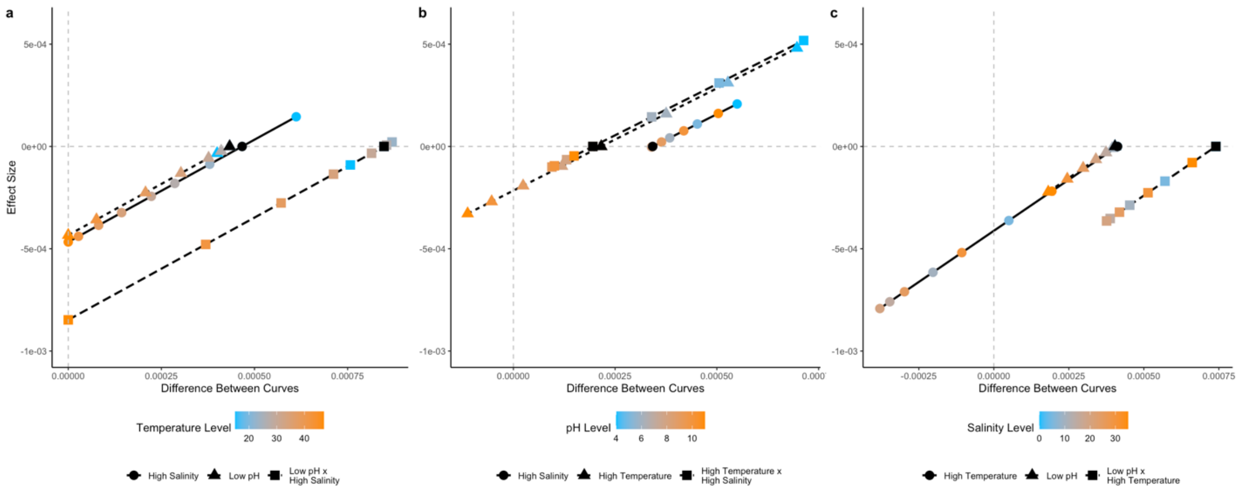

Figure S5. Linking performance curves to multi-stressor effect sizes. a-c The relationship between the additive effect size and the difference between the control performance curve and the performance curves with additional stressors along (a) temperature, (b) pH and (c) salinity gradients. Differences between curves were calculated using the fixed effects from the linear mixed effects models shown in Figure 2. Effect sizes were calculated by comparing the additive-null model prediction with the observed multi-stressor response for different levels along the gradient. Shapes represent the different additional stressors. Colours show where on the performance curve the points were taken from. Black points indicate the control level [(a) 20°C , (b) pH 7.2, (c) 0g NaCl/L].

Supplementary Tables:

Table S1. Linear mixed-effects model comparisons for temperature, pH and salinity curves. Akaike information criterion corrected for small sample size (AICc) scores to compare linear mixed effects models with various fixed and random effect structures for each stressor performance curve. All possible combinations of models were fit using maximum likelihood, starting from the most complex model (i.e. the quadratic mixed effects model with interactions between the stress factors on all parameters with random effects (M1)) to the simplest model (i.e. linear model comparing growth rate and the stress response without any interactions (M8)). Bold AICc values indicate the best fitting model (i.e. the lowest/ most negative score). “poly(Temp, 2)” indicates that a quadratic term has been fit to the temperature gradient rather than a linear term (indicated by “Temp” alone). “Stress” refers to the different stressor conditions that the curves were quantified under such as the control, two- and three-way combinations

Model	Effects	Formula	AICc
**Temperature Curves**			
**M1**	Fixed	r ~ poly(Temp, 2) * Stress	**-20192.26**
	Random	(poly(Temp, 2)|Taxa)	
**M2**	Fixed	r ~ poly(Temp, 2) + Stress	-20185.87
	Random	(poly(Temp, 2)|Taxa)	
**M3**	Fixed	r ~ poly(Temp, 2)	-20087.67
	Random	(poly(Temp, 2)|Taxa)	
**M4**	Fixed	r ~ poly(Temp, 2) * Stress	-19776.31
	Random	(1|Taxa)	
**M5**	Fixed	r ~ Temp * Stress	-19491.99
	Random	(Temp|Taxa)	
**M6**	Fixed	r ~ poly(Temp, 2) * Stress	-19198.18
	Random	NA	
**M7**	Fixed	r ~ Temp * Stress	-18831.07
	Random	NA	
**M8**	Fixed	r ~ Temp + Stress	-18831.68
	Random	NA	
**pH Curves**			
**M1**	Fixed	r ~ poly(pH, 2) * Stress	**-18051.16**
	Random	(poly(pH, 2)|Taxa)	
**M2**	Fixed	r ~ poly(pH, 2) + Stress	-18049.16
	Random	(poly(pH, 2)|Taxa)	
**M3**	Fixed	r ~ poly(pH, 2)	-18025.84
	Random	(poly(pH, 2)|Taxa)	
**M4**	Fixed	r ~ poly(pH, 2) * Stress	-17902.15
	Random	(1|Taxa)	
**M5**	Fixed	r ~ pH * Stress	-18011.25
	Random	(pH|Taxa)	
**M6**	Fixed	r ~ poly(pH, 2) * Stress	-17224.56
	Random	NA	
**M7**	Fixed	r ~ pH * Stress	-17207.13
	Random	NA	
**M8**	Fixed	r ~ pH + Stress	-17206.32
	Random	NA	
**Salinity Curves**			
**M1**	Fixed	r ~ poly(Sal, 2) * Stress	**-18464.23**
	Random	(poly(Sal, 2)|Taxa)	
**M2**	Fixed	r ~ poly(Sal, 2) + Stress	-18447.63
	Random	(poly(Sal, 2)|Taxa)	
**M3**	Fixed	r ~ poly(Sal, 2)	-18360.82
	Random	(poly(Sal, 2)|Taxa)	
**M4**	Fixed	r ~ poly(Sal, 2) * Stress	-18084.37
	Random	(1|Taxa)	
**M5**	Fixed	r ~ Sal * Stress	-18267.41
	Random	(Sal|Taxa)	
**M6**	Fixed	r ~ poly(Sal, 2) * Stress	-17488.2
	Random	NA	
**M7**	Fixed	r ~ Sal * Stress	-17432.95
	Random	NA	
**M8**	Fixed	r ~ Sal + Stress	-17435.57
	Random	NA	

Table S2. Differences in the position, width and height of performance curves between pairwise combinations of stressors across temperature gradients. Using quadratic models fit via mixed effects, comparisons were made for the linear (position), quadratic (width) and intercept (height – overall effect across temperature levels) parameters of the curve to assess changes in curve shape between temperature curves with no additional stressors (Control), curves with the addition of low pH (pH 5.5), curves with high salinity (Sal, 20g NaCl/L) and curves with a combination of pH and salinity stress (pH x Sal).

Stressor Contrasts	estimate	Std. Error	df	*t* ratio	*P* value
**Linear**					
**Control - pH**	-0.0036714	0.0015422	1801.15	-2.380585	0.017
**Control - pH x Sal**	-0.0049036	0.0015466	1801.194	-3.1706922	0.002
**Control - Sal**	-0.0045616	0.0015448	1801.576	-2.9528543	0.003
**pH - pH x Sal**	-0.0012323	0.0015488	1801.023	-0.7956464	0.426
**pH - Sal**	-0.0008902	0.0015474	1801.663	-0.5753117	0.565
**pH x Sal - Sal**	0.0003421	0.0015516	1801.658	0.220453	0.826
**Quadratic**					
**Control - pH**	-0.0131361	0.011892	1801.065	-1.1046173	0.269
**Control - pH x Sal**	-0.0226448	0.0118975	1801.087	-1.9033319	0.057
**Control - Sal**	0.0046418	0.0119256	1801.918	0.3892285	0.697
**pH - pH x Sal**	-0.0095087	0.0118995	1801.041	-0.799084	0.424
**pH - Sal**	0.0177779	0.0119278	1801.942	1.4904664	0.136
**pH x Sal - Sal**	0.0272866	0.0119333	1801.973	2.2865871	0.022
**Intercept**					
**Control - pH**	0.0002811	0.0000649	1801.16	4.3290642	<0.001
**Control – pH x Sal**	0.0006525	0.0000651	1801.089	10.0173998	<0.001
**Control - Sal**	0.0002441	0.000065	1801.327	3.7585072	<0.001
**pH – pH x Sal**	0.0003714	0.0000652	1801.058	5.6977655	<0.001
**pH - Sal**	-0.000037	0.000065	1801.486	-0.5684449	0.57
**pH x Sal - Sal**	-0.0004084	0.0000652	1801.381	-6.2616318	<0.001

Table S3. Differences in the position, width and height of performance curves between pairwise combinations of stressors across pH gradients. Using quadratic models fit via mixed effects, comparisons were made for the linear (position), quadratic (width) and intercept (height – overall effect across pH levels) parameters of the curve to assess changes in curve shape between pH curves with no additional stressors (Control), curves with the addition of high temperature (Temp, 38°C), curves with high salinity (Sal, 20g NaCl/L) and curves with a combination of temperature and salinity stress (Sal x [illegible]

Stressor Contrasts	estimate	Std. Error	df	*t* ratio	*P* value
**Linear**					
**Control - Sal**	-0.0003538	0.003138	1642.853	-0.1127444	0.91
**Control - Sal x Temp**	-0.0063064	0.0031312	1642.323	-2.0140569	0.044
**Control - Temp**	-0.009293	0.0031245	1642.168	-2.9742523	0.003
**Sal - Sal x Temp**	-0.0059526	0.0031481	1642.642	-1.8908691	0.059
**Sal - Temp**	-0.0089392	0.0031416	1642.727	-2.8454217	0.004
**Sal x Temp - Temp**	-0.0029865	0.0031349	1642.182	-0.9526852	0.341
**Quadratic**					
**Control - Sal**	0.0025482	0.0030753	1643.49	0.8286055	0.407
**Control - Sal x Temp**	0.0036216	0.0030539	1642.528	1.185902	0.236
**Control - Temp**	0.0016077	0.0030554	1642.503	0.5261802	0.599
**Sal - Sal x Temp**	0.0010734	0.0030772	1643.229	0.3488333	0.727
**Sal - Temp**	-0.0009405	0.0030796	1643.559	-0.3053909	0.76
**Sal x Temp - Temp**	-0.0020139	0.0030578	1642.438	-0.6586178	0.51
**Intercept**					
**Control - Sal**	0.0004139	0.0000792	1642.484	5.2232864	<0.001
**Control – Sal x Temp**	0.0002669	0.0000792	1642.216	3.3704618	<0.001
**Control - Temp**	0.0002141	0.0000793	1642.248	2.7004446	0.007
**Sal – Sal x Temp**	-0.000147	0.0000795	1642.546	-1.8476665	0.065
**Sal - Temp**	-0.0001998	0.0000796	1642.758	-2.5087415	0.012
**Sal x Temp - Temp**	-0.0000528	0.0000796	1642.176	-0.6637454	0.507

Table S4. Differences in the position, width and height of performance curves between pairwise combinations of stressors across salinity gradients. Using quadratic models fit via mixed effects, comparisons were made for the linear (position), quadratic (width) and intercept (height – overall effect across salinity levels) parameters of the curve to assess changes in curve shape between salinity curves with no additional stressors (Control), curves with the addition of high temperature (Temp, 38°C), curves with low pH (pH 5.5) and curves with a combination of temperature and pH stress (pH x Temp).

Stressor Contrasts	estimate	Std. Error	df	*t* ratio	*P* value
**Linear**					
**Control - pH**	-0.0026665	0.0026871	1652.018	-0.9923455	0.321
**Control - pH x Temp**	-0.000947	0.0026913	1652.054	-0.3518765	0.725
**Control - Temp**	-0.0026233	0.002697	1652.071	-0.9726552	0.331
**pH - pH x Temp**	0.0017195	0.0026895	1652.032	0.6393481	0.523
**pH - Temp**	0.0000433	0.0026953	1652.064	0.0160517	0.987
**pH x Temp - Temp**	-0.0016763	0.0026995	1652.08	-0.6209434	0.535
**Quadratic**					
**Control - pH**	-0.0009058	0.0024339	1652.14	-0.3721611	0.71
**Control - pH x Temp**	0.0044742	0.0024417	1652.199	1.8324279	0.067
**Control - Temp**	0.009353	0.0024373	1652.176	3.8374298	<0.001
**pH - pH x Temp**	0.00538	0.0024392	1652.058	2.2056471	0.028
**pH - Temp**	0.0102588	0.002435	1652.104	4.2131192	<0.001
**pH x Temp - Temp**	0.0048788	0.0024424	1652.089	1.997588	0.046
**Intercept**					
**Control - pH**	0.0003301	0.0000707	1652.074	4.6671066	<0.001
**Control – pH x Temp**	0.0005141	0.0000709	1652.172	7.2543433	<0.001
**Control - Temp**	-0.0000869	0.0000709	1652.123	-1.2257626	0.22
**pH – pH x Temp**	0.000184	0.0000709	1652.098	2.5958889	0.01
**pH - Temp**	-0.000417	0.0000709	1652.072	-5.8835526	<0.001
**pH x Temp - Temp**	-0.000601	0.000071	1652.085	-8.4648185	<0.001

Table S5. Changes in multiple stressor interactions across gradients and between taxa. Comparing full and reduced generalised additive model (GAM) fits investigating relationships between temperature, pH and salinity gradients and interaction effect sizes (Hedges' *d*). Significance of parameters within the model was determined using Akaike information criterion corrected for small sample size (AICc). This was carried out for each combination of stressors along each of the stressor gradients. Full models allowed the intercepts and spline shapes to vary by taxon Id along the gradients. The lower the AICc, the better the model fit.

Stressors	Model Parameters	AICc
**Temperature Curves**		
**Temperature x Salinity**	Taxa, s(Temp Level, by = Taxa)	532.918
	Taxa, s(Temp Level)	535.601
	s(Temp Level)	537.9816
	Taxa	537.184
	Null	539.0946
**Temperature x pH**	Taxa, s(Temp Level, by = Taxa)	434.8071
	Taxa, s(Temp Level)	461.4929
	s(Temp Level)	459.8171
	Taxa	461.4583
	Null	459.8156
**Temperature x pH x Salinity**	Taxa, s(Temp Level, by = Taxa)	521.645
	Taxa, s(Temp Level)	546.6796
	s(Temp Level)	552.6395
	Taxa	546.7385
	Null	552.6672
**pH Curves**		
**pH x Salinity**	Taxa, s(pH Level, by = Taxa)	382.3519
	Taxa, s(pH Level)	451.8107
	s(pH Level)	467.5411
	Taxa	454.1896
	Null	468.7098
**pH x Temperature**	Taxa, s(pH Level, by = Taxa)	348.7832
	Taxa, s(pH Level)	373.2709
	s(pH Level)	368.9133
	Taxa	381.1835
	Null	374.2283
**pH x Temperature x Salinity**	Taxa, s(pH Level, by = Taxa)	388.983
	Taxa, s(pH Level)	430.1077
	s(pH Level)	489.2083
	Taxa	442.5807
	Null	492.9877
**Salinity Curves**		
**Salinity x Temperature**	Taxa, s(Salinity Level, by = Taxa)	384.8893
	Taxa, s(Salinity Level)	431.9487
	s(Salinity Level)	503.6804
	Taxa	431.9446
	Null	503.6804
**Salinity x pH**	Taxa, s(Salinity Level, by = Taxa)	359.1619
	Taxa, s(Salinity Level)	416.6281
	s(Salinity Level)	424.8625
	Taxa	417.9923
	Null	425.622
**Salinity x pH x Temperature**	Taxa, s(Salinity Level, by = Taxa)	346.6148
	Taxa, s(Salinity Level)	405.0941
	s(Salinity Level)	456.4406
	Taxa	405.2841
	Null	456.4406

Table S6. Stressor combinations and levels for each of the temperature, pH and salinity gradients.

Gradient	Stressors	Temperature (°C)	Salinity (g NaCl/L)	pH
**Temperature**	Control	-	0	7.2
	pH	-	0	5.5
	Salinity	-	20	7.2
	pH x Salinity	-	20	5.5
**pH**	Control	20	0	-
	Temperature	38	0	-
	Salinity	20	20	-
	Temperature x Salinity	38	20	-
**Salinity**	Control	20	-	7.2
	Temperature	38	-	7.2
	pH	20	-	5.5
	pH x Temperature	38	-	5.5
